# Within-population variability in coral heat tolerance indicates climate adaptation potential

**DOI:** 10.1098/rspb.2022.0872

**Published:** 2022-08-31

**Authors:** Adriana Humanes, Liam Lachs, Elizabeth A. Beauchamp, John C. Bythell, Alasdair J. Edwards, Yimnang Golbuu, Helios M. Martinez, Paweł Palmowski, Achim Treumann, Eveline van der Steeg, Ruben van Hooidonk, James R. Guest

**Affiliations:** ^1^ School of Natural and Environmental Sciences, Newcastle University, Newcastle upon Tyne, UK; ^2^ Palau International Coral Reef Center, Koror, Palau; ^3^ Cooperative Institute for Marine and Atmospheric Studies, Rosenstiel School of Marine and Atmospheric Science, University of Miami, Miami, FL 33149, USA; ^4^ Atlantic Oceanographic and Meteorological Laboratory, National Oceanic and Atmospheric Administration, Miami, FL 33149, USA

**Keywords:** phenotypic response, coral bleaching, heat tolerance, climate change

## Abstract

Coral reefs are facing unprecedented mass bleaching and mortality events due to marine heatwaves and climate change. To avoid extirpation, corals must adapt. Individual variation in heat tolerance and its heritability underpin the potential for coral adaptation. However, the magnitude of heat tolerance variability within coral populations is largely unresolved. We address this knowledge gap by exposing corals from a single reef to an experimental marine heatwave. We found that double the heat stress dosage was required to induce bleaching in the most-tolerant 10%, compared to the least-tolerant 10% of the population. By the end of the heat stress exposure, all of the least-tolerant corals were dead, whereas the most-tolerant remained alive. To contextualize the scale of this result over the coming century, we show that under an ambitious future emissions scenario, such differences in coral heat tolerance thresholds equate to up to 17 years delay until the onset of annual bleaching and mortality conditions. However, this delay is limited to only 10 years under a high emissions scenario. Our results show substantial variability in coral heat tolerance which suggests scope for natural or assisted evolution to limit the impacts of climate change in the short-term. For coral reefs to persist through the coming century, coral adaptation must keep pace with ocean warming, and ambitious emissions reductions must be realized.

## 1. Introduction

Climate change is having profound impacts on marine ecosystems due to an increased frequency and severity of marine heatwaves, including mass mortalities, shifts in species distributions and altered ecological function and ecosystem services [1]. Heat stress can alter organism physiology, behaviour and fitness, and in extreme cases directly cause mortality. Heat tolerance is often considered as a characteristic trait of populations, species or genera [2]. However, large trait differences are present among individuals within a population and this can arise through genetic [3], epigenetic [4] and environmental effects [5]. Heat stress is predicted to worsen in the coming decades, but it is unclear by how much and to what extent organisms will adapt. Quantifying the extent of intrapopulation variability of heat tolerance (i.e. the combined phenotypic responses that dictate survivorship to a marine heatwave) is fundamental to predicting impacts of ocean warming, particularly for long-lived sessile marine invertebrates that cannot migrate to escape warming and must adapt to avoid extirpation.

Corals are highly susceptible to relatively small (approx. 1–2°C) but prolonged increases in sea temperatures above those typical of the hottest month [6]. Heat stress results in bleaching, the breakdown of the symbiosis between the coral host and its symbiotic algal counterparts (Symbiodiniaceae). This response depends on both the magnitude and duration of heat stress, typically expressed as degree heating weeks (DHW,°C-weeks) and calculated by accumulating positive temperature anomalies relative to a coral stress baseline over a 12-week moving window [7]. The likelihood of different corals surviving an individual warming event (i.e. relative heat tolerance, RHT) depends upon coral species [8], host genome [9], algal symbiont species composition [10], the prokaryote microbiome [11], local environmental conditions [12] and bleaching history [13]. With increasing mass coral bleaching events across pantropical spatial scales due to marine heatwaves, the persistence of some coral taxa and ecosystems under projected climate change scenarios is uncertain. As a result, there have increasingly been calls to accelerate natural adaptation through active interventions such as assisted evolution [14,15] which aims to increase the frequency of specific adaptive traits across a population to improve their future survivorship [16,17]. Both natural and artificial selection depend in part on there being sufficient standing genetic variation in adaptive traits such as RHT.

Here we quantify heat tolerance variability within a coral population and address the challenge of defining and identifying heat-tolerant corals. Given the extent of projected ocean warming and coral bleaching conditions [18], heat tolerance is the trait most-likely to bolster coral reef resilience under climate change [9,19–22]. However, heat tolerance is a complex multivariate trait. It is complex because it is influenced by many genes and environmental factors [9], and multivariate because no single phenotypic variable can account for the variety of responses to different heat stress profiles [23]. Indeed, the type of response that an organism exhibits may vary throughout the time course of an event, and an individual that appears more heat tolerant at the start of an exposure may appear susceptible at a later stage. In order to comprehensively account for such exposure–time effects, we define heat tolerance as the combined bleaching and mortality response to extended heatwave conditions. This definition accounts for varying bleaching and mortality responses during the time course of a temperature stress [24]. To allow a measure of heat tolerance to reflect the duration of marine heatwaves and mass bleaching events (weeks to months) [25], we used a 30-day long-term [26] experimental temperature profile. This resulted in levels of accumulated heat stress broadly equivalent to previous mass bleaching and mortality events in Palau (electronic supplementary material, S5). To contextualize the differences observed in heat tolerance among individuals of the population, we extrapolated the results into projected timings of onset of annual mass bleaching and mortality conditions under two contrasting climate change scenarios.

## 2. Results

### (a) Overall response to heat stress

The heat stress conditions used during the experiment were sufficient to elicit a response in most of the fragments (96%, *n* = 544). The response varied markedly from healthy (no visible response; seen in 4% of the fragments) to partially bleached, fully bleached, partially dead or completely dead at the end of the experiment (30 days). The visible condition of the coral fragments showed a similar range and time course of responses to those that have been observed in mass bleaching events over recent decades. Bleaching was observed in almost three quarters (72%) of the heat-stressed fragments, which on average spent 6 days bleached, whereas 13% changed their health status directly from apparently healthy to dead between inspections. At the colony level (*n* = 102), the mean time to effect (partially bleached, bleached, partial mortality or death) was 21 days or 7°C-weeks, equivalent to Alert Level 1 of the NOAA Coral Reef Watch Bleaching Alert System [7].

### (b) Categorical classification of heat tolerance

**Figure 1.**
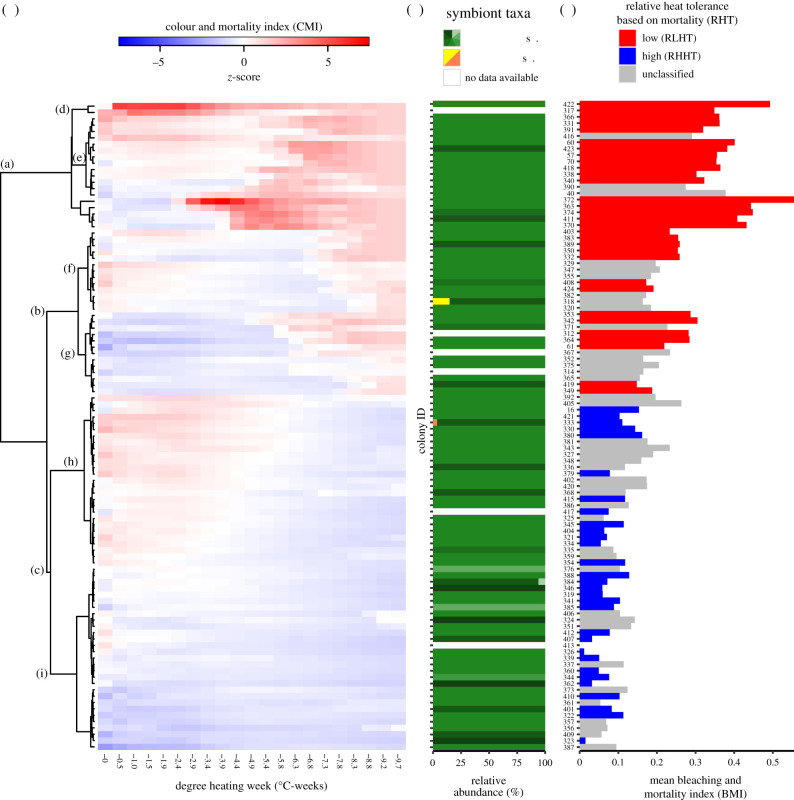
Heat stress tolerances of 102 colonies exposed to a temperature stress event of long-term duration (30 days) and taxonomic composition of their associated Symbiodiniaceae. (*a*) Profile of CMI (rows) against accumulated heat stress (columns) expressed in degree heating weeks (DHW). (*b*) Symbiodiniaceae populations associated with sampled corals. Bars show post quality control (post-QC) ITS-2 sequences for each coral colony. For complete disclosure of the Symbiodiniaceae taxonomic resolution refer to electronic supplementary material, S3. (*c*) Bar chart for mean BMI ranked according to colonies position in the heat map in (*a*). Colours indicate RHT of the colonies, with relative low heat-tolerant colonies indicated in blue, relative high heat-tolerant colonies indicated in red, and unclassified colonies in grey. (Online version in colour.)

A mortality-based classification of the RHT assigned a third of the colonies (29%) as highs (relatively high heat tolerance, RHHT, *p* < 0.05) and a similar large proportion (30%) as lows (relatively low heat tolerance, RLHT, *p* < 0.05), with the remaining 40% unclassified (figure 1*c*). While the dosage of heat required to elicit a bleaching and mortality response varied substantially between lows and highs, there was little difference at bleaching onset between lows (6.6°C-weeks) and highs (7.7°C-weeks). However, all replicates from lows were dead by 7.3°C-weeks whereas all those from highs were still alive at 9.4°C-weeks. These results were also reflected in colony mean bleaching and mortality index (BMI) values (range = 0–1), with colonies classified as highs having a significantly lower mean BMI (0.08 ± 0.04 s.d.) than those classified as lows (0.32 ± 0.10 s.d., Wilcoxon rank test *W* = 928, *p* < 0.001) (figure 1*c*).

### (c) Phenotypic responses to heat stress form distinct groups

Clusters of distinct phenotypic response types are apparent from colour and mortality index (CMI) profiles (figure 1*a*), showing finer resolution health status changes than could be detected by eye using BMI profiles (electronic supplementary material, S1A). The imaging basis of CMI can detect colour changes long before bleaching is visually evident to the observer (electronic supplementary material, S2). In the CMI profiles, colonies classified as lows (RLHT) and with the highest BMI values clustered together in two major groups (nodes a and b, figure 1*a*), whereas colonies classified as highs (RHHT) clustered in one major group (node c, figure 1*a*). The comparison of profiles among groups revealed different types of phenotypic responses to the heat stress. For colonies classified with low heat tolerance at least three distinctive response trajectories were apparent: (i) nodes d and e, corresponding to colonies with the biggest changes in their colour and survivorship status (i.e. CMI) from the beginning till the end of the stress in comparison to colonies in any other node, (ii) node f, grouping colonies with the greatest changes in their CMI at the beginning of the stress, but as DHW increased these changes were not as pronounced in relation to colonies in other nodes (i.e. between approx. 3.9 and approx. 7.8°C-weeks for nodes d and g), and, (iii) node g, with colonies that did not show a substantial change in CMI until corals were exposed approximately to 5.8°C-weeks. Correspondingly, high colonies exhibited two response types: (i) node h, grouping colonies that were paler (had relatively higher CMI values) at the beginning of the exposure but as the stress progressed these changes did not lead to bleaching or mortality, while (ii) node i, corresponding to colonies that did not show any major changes in their relative status (i.e. *z*-scored CMI) throughout the heat stress.

### (d) Symbiodiniaceae community composition and heat tolerance

The Symbiodiniaceae community composition was dominated by a single ITS-2 type profile, with 77% (*n* = 102) of the examined colonies containing *Cladocopium* spp. (C40-C3-C115-C40 h) as the major component. Equal numbers (*n* = 20) of colonies identified as highs and lows contained only this symbiont-type profile. A small proportion of the remaining colonies (9%) had unique type profiles (seven highs and two lows), but they were still dominated by *Cladocopium* spp. (C40 variant). Three colonies contained two distinct symbiont-type profiles, with the additional presence of minor components of either *Cladocopium* spp. (C15 h variant *n* = 1) or *Durisdinium* spp. (either D1-D4-D1c-D17d-D1r-D17c-D17e variant, *n* = 1; or D1-D4-D17d-D4c-D17e-D1r-D17c, *n* = 1; figure 1*b*, electronic supplementary material, S3). No significant relationship was observed between the taxonomic composition of the Symbiodiniaceae community and the RHT of the colonies (table 1; electronic supplementary material, S4).

**Table 1.** Percentage of colonies within each dominant ITS-2 type profile according to their RHT classification (relative high heat tolerant, relative low heat tolerant and unclassified) based on mortality.

ITS-2	high (RHHT) (%)	low (RLHT) (%)	unclassified (%)	total (%)
C40-C3-C115-C40 h	20	20	37	77
C40-C3-C40j	2	4	3	9
C40-C40i-C3	3	0	1	4
C40-C3-C40i-C40j	0	2	2	4
C40-C15 h-C3-C115-C40 h	1	0	1	2
C40/C3	1	0	0	1
C40-C3-C40j and C15	1	0	0	1
C40-C3-C40j and D1-D4-D1c-D17d-D1r-D17c-D17e	1	0	0	1
C40-C3-C40j and D1-D4-D17d-D4c-D17e-D1r-D17c	0	0	1	1

### (e) Quantification of intrapopulation variation in heat tolerance

**Figure 2.**
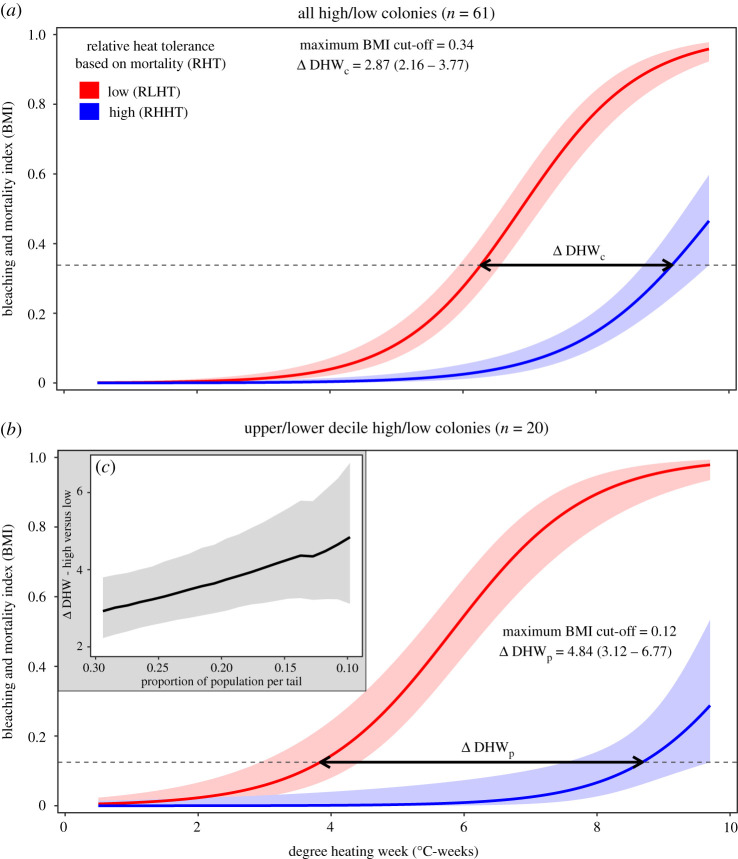
Quantification of intrapopulation variation in heat tolerance between colonies classified as having low or high RHT based on mortality (RHHT—blue/lower line and RLHT—red/upper line, respectively). Variation is expressed by differential relationships between DHW and the BMI (mean ± 95% confidence intervals). (*a,b*) Intrapopulation heat tolerance variability is shown as the difference in heat stress tolerated between highs and lows (ΔDHW ± 95% confidence intervals, bold horizontal arrow) for a given phenotypic response (dashed line, the maximum horizontal cut-off that includes all confidence intervals). (*a*) A conservative estimate of heat tolerance variability (DHW_c_) was based on the full high/low groups (*n* = 61 colonies, ΔDHW_c_ = 2.87°C-weeks at BMI = 0.34). This is less than (*b*) the realized population-level heat tolerance variability (ΔDHW_p_) quantified from decile population subsamples (*n* = 20 colonies, ΔDHW_p_ = 4.84°C-weeks at BMI = 0.12). (*c*) As colonies are subsampled further into the population extremes or tails, the quantified intrapopulation variability in heat tolerance increases beyond 4°C-weeks. (Online version in colour.)

Accumulated heat stress levels exceeding 8°C-weeks (operational DHW, NOAA Coral Reef Watch) are typically considered sufficient to cause severe bleaching and mortality [7]. Using a conservative estimate of heat tolerance variability (*Δ* DHW_c_) based on lows (all five fragments per colony in temperature stress tanks dead at the end of the exposure; *n* = 31 colonies) and highs (all five fragments in temperature stress tanks alive at the end of exposure; *n* = 30 colonies), highs were able to withstand an additional 2.87°C-weeks of heat stress (2.16–3.77°C-weeks 95% confidence interval, figure 2*a*) compared to lows at the highest equivalent BMI reached. More substantial differences in heat tolerance (*Δ* DHW_p_) were detectable by examining the population tails. There was a difference of 4.84°C-weeks (3.12–6.77°C-weeks 95% confidence intervals, figure 2*b*) between the upper and lower deciles of the population, measured at the highest equivalent BMI reached. For onset of bleaching responses (i.e. low BMI values), corals in the most-tolerant decile required double the heat stress dosage (approx. 8.5 DHW) compared with those in the least-tolerant decile (approx. 4 DHW) (figure 2*b*).

### (f) Implications of variability under climate change

**Figure 3.**
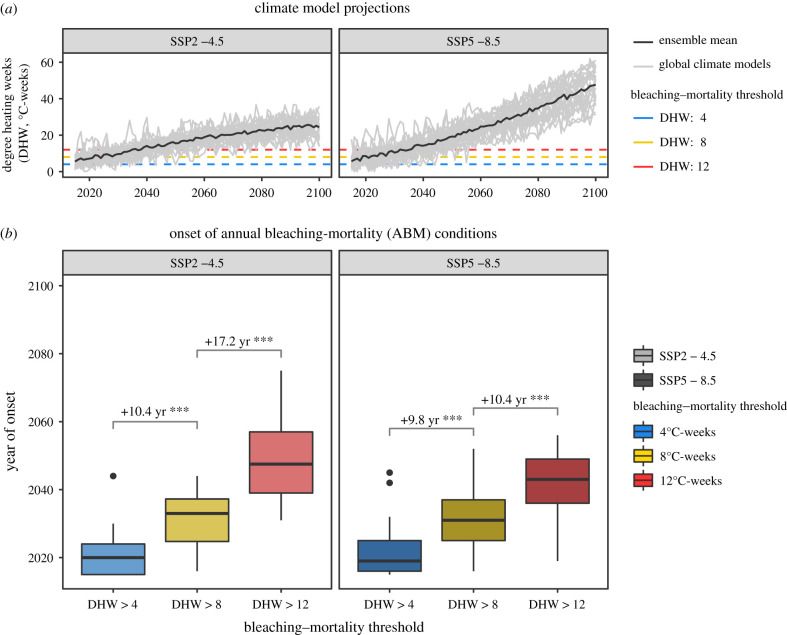
(*a*) Coral bleaching heat stress (Degree Heating Weeks, DHW) at Mascherchur reef was projected from 28 global climate models from the CMIP6 between two global shared socioeconomic pathways: SSP2-4.5 (meeting 150% of Paris Agreement pledges) and SSP5–8.5 (worst-case scenario, growing world economy heavily dependent on fossil fuels). Three bleaching-mortality thresholds (4°C, 8°C and 12°C-weeks; blue/lower dashed line, yellow/middle dashed line and red/upper dashed line, respectively) were implemented to derive projections of bleaching-mortality conditions for specific levels of heat tolerance. The difference among these thresholds is broadly comparable to the level of intrapopulation variation in heat tolerance quantified through experimentation. (*b*) The difference in timing of onset of annual bleaching mortality (ABM) conditions are shown between SSP2 and SSP5, and among the different levels of coral heat tolerance (see thresholds). Only statistically significant (***p < 0.001) comparisons of ABM onset are shown, based on an LMM and Tukey test. (Online version in colour.)

Based on future projections of coral bleaching heat stress conditions (DHW) from 28 global climate models, the study reef is projected to experience further warming throughout the coming century (figure 3*a*). An empirical difference of 4.84°C-weeks was detected between the most and least-tolerant decile of the population, which is greater than an entire categorical shift in the NOAA bleaching alert levels (Bleaching Warning: 0 < DHW less than 4, Bleaching Alert Level 1: 4 ≤ DHW < 8, Bleaching Alert Level 2: DHW ≥ 8). Therefore, we simulated coral heat tolerances in future projections with bleaching-mortality thresholds of 4°C, 8°C and 12°C-weeks (figure 3*b*). Broadly, simulating corals with a 4°C-weeks higher heat tolerance translated to an additional 10 years until the onset of annual bleaching–mortality (ABM) conditions, irrespective of the emission scenario (SSP2–4.5 and SSP5-8.5 [27]). However, under the ambitious SSP2-4.5 scenario, simulations suggested that the most heat-tolerant corals (that survive up to 12°C-weeks) gained an additional 17 years until onset of ABM conditions compared to less-tolerant corals (that survive up to 8°C-weeks).

### (g) Discussion

Despite a growing understanding that coral heat tolerance may vary spatially [13], temporally [28], and among symbiont [29] and host taxa [24], relatively few studies [30–41] have quantified within-population variability of heat tolerance. Among these previous studies, most have focused solely on the bleaching responses to temperature stress (usually short-term acute stress), with none addressing both bleaching and mortality to heat stress conditions equivalent to a marine heatwave. Addressing this knowledge gap is critical to estimate the capacity of corals to adapt to predicted warming. In this study, we demonstrate that intrapopulation heat tolerance is highly variable, with the most-tolerant 10% of colonies able to withstand an additional 4.8°C-weeks of heat stress compared to the least-tolerant 10% before exhibiting equivalent BMI response. We also estimate the impact that climate mitigation can have on coral population persistence given their heat tolerance variability. For the highest level of coral heat tolerance simulated here (12°C-weeks), an additional 7 years until onset of ABM conditions was achieved by moving from SSP5-8.5 (+10 years) to SSP2 (+17 years, figure 3*b*). While our empirical experiments show substantial intrapopulation variability in heat tolerance, our projections based on simulated heat tolerance suggest that the resulting differences are only able to substantially bolster reef resilience if there is strong action on climate change.

Characterizing coral heat tolerance using temperature stress assays is challenging. Firstly, no simple metric (e.g. physiological variables, visual bleaching status, mortality) can summarize the response of the coral holobiont to heat stress. Secondly, no heat stress events are the same and the amount of temperature stress experienced is a function of duration, intensity and prior exposure, all of which vary stochastically at a given location. Thirdly, the logistical constraints of maintaining healthy corals or their fragments in mesocosms and subjecting them to controlled temperature rises for sustained periods (e.g. weeks to months), has resulted in only a few studies attempting to emulate thermal trajectories associated with past mass bleaching events (greater than or equal to 8 DHW) [42]. Finally, heat tolerance is a complex phenotypic trait (non-Mendelian trait influenced by environmental and genetic factors), hence responses will be influenced by other factors including health status prior to the onset of the heat stress [43]. Most studies estimate interpopulation differences in heat tolerance among corals from thermally distinct environments, such that higher levels of tolerance are associated with temperature variability and warmer reefs [21,44], while less attention has been given to intrapopulation variability. Common metrics used to quantify coral heat tolerance under laboratory conditions are associated with physiological changes in the symbiont community when exposed to short-term [26] heat stress (i.e. pigment concentrations, algal symbiont density, reduction of the photosystem II efficiency, microbiome community composition). However, there is little available evidence that such variables measured in short-term stress assays predict colony mortality during mass bleaching events, indeed previous studies have reported that bleaching status is not necessarily a strong predictor of later mortality [24]. Results from the present study, emulating a long-term heat stress, showed categorical variation in responses within the population, with early responses (e.g. bleaching, figure 1*a*) not always predicting coral mortality. To project future population persistence and natural or assisted evolution, more realistic temperature stress experiments (i.e. long-term) are needed, with individual mortality as the endpoint. Moreover, we should be cautious when using results from temperature stress experiments to predict how corals will respond to natural heatwaves, as responses under mesocosm conditions will likely differ from individual colony responses in their natural habitat with abiotic (e.g. oxygen concentration, light, nutrient enrichment, water flow) and biotic (e.g. predation, food availability, competition, diseases) factors potentially interacting simultaneously. Future research should consider validating experimental heat tolerance assignments by tracking the same colonies during natural bleaching events.

Here we use the term RHT to compare the heat resistance of individual colonies within the context of a specific temperature stress experiment. Importantly, this RHT may change depending on the context and be defined by the identity of the colonies present in the exposure and the heat stress profile used. Furthermore, corals will exist along a continuum of heat tolerance phenotypes, where some colonies have higher resistance to heat stress than others. Therefore, terms like ‘super coral', ‘resistant coral' or ‘tolerant coral’ lack biological significance [45] as the tolerance of a colony is not absolute and will be relative to the phenotypic response of other individuals for the given stress. This study quantitatively estimates RHT based on individual bleaching and mortality responses as a function of heat stress (i.e. DHW). The estimation of RHT is useful to: (i) understand the mechanisms underpinning variation in heat tolerance, (ii) select colonies with desirable phenotypic traits for restoration and conservation initiatives (i.e. establishment of nurseries, fragment transplantation, selective breeding) assuming there is a heritable genetic component to RHT, (iii) compare individual heat tolerances within and among populations, as well as among species to estimate population adaptive potential, design management and conservation plans to improve their resilience under future ocean warming scenarios.

In this study, in order to overcome issues associated with single metric variable responses we used a combination of variables (i.e. colour change, health status, pigment concentration, zooxanthellae density, mortality) to evaluate the phenotypic response of corals to heat stress. The different analytical approaches provided complementary information about individual resistance to heat stress. By using a categorical RHT classification combined with a continuous bleaching-mortality index, we were able to quantify and characterize differences in heat resistance among colonies (figure 2). Our results show clear evidence that RHT of individual corals varies significantly at the population level. There were several distinct bleaching trajectories among colonies with similar RHT (figure 1*a*) that may represent different physiological response mechanisms or strategies [46]. Heat tolerance is a composite multivariate phenotypic trait with multiple pathways to improved trait performance within an individual or population (e.g. the type and amount of energy reserves [43], constitutive immunity [47], host and symbionts genotype [33,48] and their interaction [47], gene frontloading [36], Symbiodiniaceae community diversity [49], switching [50] and reshuffling [29,51], microbiome turnover [11]). It was beyond the scope of this study to investigate the underlying causes of variation in heat tolerance, which would require sampling before, during and after the exposure. However, the Symbiodiniaceae community prior to the heat stress was dominated by a single ITS-2 type profile (C40-C3-C115-C40 h) which was equally dominant in highs and lows. Although flexibility [49] and diversity [52] of symbionts have been associated with heat tolerance, in this intrapopulation study, initial symbiont type does not explain the observed variation in RHT.

Our results show that wild coral populations have substantial variation in heat tolerance, suggesting a reservoir of adaptive potential to resist temperature stress due to climate change [53]. Importantly, we do not explicitly model coral adaptation in this study. Provided that the variation we measured has a genetic basis, is heritable and is not associated with resource trade-offs that compromise population fitness, then our results suggest potential for adaptation through natural or assisted evolution. Furthermore, previous mass bleaching events might have already increased the frequency of high tolerance colonies at the population level [54] through processes like selective sweeps [20]. Restoration efforts that include assisted evolution are being considered as interventions to help coral populations to adapt to climate change, but with a focus on assisted migration or assisted gene flow between populations from different thermal environments [15,19,20]. Our study suggests that assisted evolution restoration initiatives could be effective using local colonies of the target population instead. Sourcing more heat-tolerant corals from within populations has several potential advantages as it reduces: (i) the likelihood of maladaptation to other local environmental variables [55], (ii) the risk of inadvertently selecting different genetic variants or sub-species [56], (iii) the probability of modifying genetic structures of native local populations [57] (however see Jones [58]), (iv) the introduction of pathogens or parasites that could compromise the abundance and health of local species [59], (v) the ethical [60], legal [60] and cost [61] implications associated with translocating individuals among populations.

Globally, acroporid coral populations are projected to be lost by the middle of this century, even under moderate emissions scenarios [62–64]. However, such projections have not accounted for variability in coral heat tolerance. Given that acroporid corals typically have high population turnover rates [65], this variability could lead to rapid adaptation [53]. Here we show that the levels of variation in heat tolerance within a single *Acropora* population, can result in a difference of at least 10–17 years until onset of ABM conditions between the most and the least-tolerant corals. Since severe bleaching conditions (8°C-weeks) are projected to occur annually by 2032 within this population, such an increase could almost double the remaining time that some corals have until facing annual mass bleaching conditions. The additional 10–17 years provides scope for population adaptation to occur through either natural selection or assisted evolution. While such a time frame will not mitigate against the worst-case emissions scenario (SSP5–8.5), it may allow coral reef ecosystems to adapt to warming, and the lagged effects of climate mitigation to take effect (i.e. carbon emission reduction and carbon sequestration). While our projections are based on the current range of DHW tolerance (which has arisen through historical adaptation), they do not account for future adaptation. We also have not accounted for adaptation of the symbiont, or symbiont shuffling, which have potential to increase temperature tolerance further [62]. Additionally, coral larvae may be supplied through open dispersal from populations with thermal histories that foster local coral heat tolerance [66]. Together these factors suggest that the additional 10–17 years for more heat-tolerant corals in a population is likely an underestimate, offering optimism for the persistence of coral reefs in the short-term (i.e. until 2050). For longer-term persistence, urgent action on climate change is still needed.

In conclusion, this study quantified substantial variability in heat tolerance in a single coral population. Although previous studies suggest that such variability may be common [30–32], it has rarely been quantified at the population scale. Our results improve the understanding on the extent of the phenotypic pool available for both natural and artificial selection. Coupling this knowledge with future climate projections provides policy relevant metrics that can directly inform coral reef conservation and restoration actions.

## 3. Material and methods

### (a) Collection of coral fragments and temperature stress experimental set-up

Heat stress experiments were carried out at the Palau International Coral Reef Center (PICRC) in the Republic of Palau located in Western Pacific Ocean. The reef-building coral *Acropora digitifera* (Dana, 1846), a widely distributed and abundant species on shallow reefs throughout the Indo-West Pacific, was used as the study organism. Its digitate morphology facilitates fragment removal for conducting stress assays. The source site for all colonies was a shallow, exposed patch reef (Mascherchur, 07°17′ 29.3″ N; 134°31′ 8.00″ E), where *A. digitifera* is abundant at depths ranging between 0 and 4 m. The historic sea surface temperature (SST) profile from Mascherchur reef for the period 1985–1995 (5 km grid cell from CoralTemp v3.1) ranged from 27.5°C to 29.7°C (daily average) with extreme high values reaching 30.7°C (upper 95% quantile, electronic supplementary material, S5), the hottest periods of the year being May, June and November. Mass coral bleaching in Palau has only been reported in 1998 and 2010 [67–69]. In Mascherchur, this was associated with maximum daily SST of 31.1°C and 30.7°C, respectively. Both heatwaves resulted in levels of accumulated heat stress of greater than or equal to 6°C-weeks, which have not been reached since (electronic supplementary material, S5).

To determine the RHT of 102 visibly healthy adult colonies a 30-day temperature stress experiment was performed. Between 9 and 13 April 2019, at least six fragments were excised from each colony and transported by boat in 50 l seawater tanks to PICRC (approx. 20 min boat travel time). On the day of collection fragments were glued to aragonite substrata (approx. 20 mm diameter, Oceans Wonders LLC) with ethyl cyanoacrylate gel (Coraffix gel) and mounted into labelled plastic holders that were attached to plexiglass racks (electronic supplementary material, S7). Racks were placed into one of six experimental tanks having one fragment per colony in each replicate tank. Relevant National and State permits were obtained for the collections (National Marine Research Permits: RE-19-08).

The temperature stress exposure started on 19 April, allowing a minimum of a 6-day recovery after fragment collection. Two temperature levels were used: (a) ambient seawater temperature conditions (29.09 ± 0.97°C, one procedural control tank with one fragment from each colony), and (b) heat stress conditions where temperature was increased gradually from approximately 29°C to approximately 32.5°C in a 30-day period (+1°C on day 1, 4 and 8, and 0.5°C on day 21, five replicate tanks each with one fragment from each colony). If a fragment from a colony died in the procedural control tank, we considered it an indication of handling effects for that colony, so all fragments from that colony in the stress tanks were removed from the experiment and the colony was not assigned a heat tolerance score. For some colonies, an additional fragment was added for pigment concentration and symbiont density analysis. The level of accumulated heat stress within each tank was monitored using an adaptation of the degree heating week (DHW) algorithm. DHW reflects the intensity and duration of heat stress events and is commonly linked to coral bleaching responses [7,25]. Daily DHW values were calculated as the rolling sum over a 12-week (84-day) period of positive SST anomalies that are greater than 1°C above NOAA's CoralTemp v3.1 1985–2012 baseline (maximum of monthly means SST climatology, MMM), then divided by seven to achieve weekly units. An adjustment of the stress threshold temperature was performed to ensure that heat stress levels in the tank experiment were comparable to heat stress levels measured in the commonly used satellite data (electronic supplementary material, S6). Since reported mass coral bleaching and mortality in 1998 and 2010 [67–69] in Palau were associated with DHW ≥ 6°C-weeks (https://coralreefwatch.noaa.gov/product/vs/gauges/palau.php), we had an *a priori* expectation for coral bleaching and mortality in the tank experiment to occur at similar DHW levels.

To reflect a population mortality rate of 50% from a mass bleaching event, when half of the fragments in each tank had died, the experiment was ended for that tank. The status of each fragment was visually inspected by the same observer every 3–5 days until first visual signs of change in pigmentation (day 10), after which monitoring was conducted daily or every other day. The status of each fragment was ranked as: 0) healthy (no signs of discoloration or mortality), (1) partially bleached (when less than 50% of the tissue was discoloured), (2) bleached (more than 50% of the tissue was discoloured), (3) partial mortality (when less than 30% of coral tissue was lost) or, (4) dead (30–100% of coral tissue was lost). Fragments were coded with consecutive numbers so that the colony ID was unknown to the observer. Alongside the status monitoring, digital images of the fragments were taken using an Olympus TG5 camera. For imaging, the mounted fragments together with a colour checker (X-Rite Color Checker Passport Photo), were placed in a 10 l tank (electronic supplementary material, S7) containing seawater at the corresponding treatment temperature. The tank was placed inside a photography light box (Amzdeal Foldable Photo Studio 16 × 16 Inch LED Lighting Box) to minimize light variability between images, and the automatic shooting setting in the camera was used.

### (b) Determination of heat stress categories

Four different but complementary approaches were used to determine RHT of each colony:

#### (i) Mortality at the end of the experiment

RHT was determined by the endpoint mortality, 30 days after the first temperature increase. Colonies with all replicate stressed fragments alive were considered to have relatively high heat tolerance (RHHT), whereas colonies with all stressed fragments dead (100% mortality) were classified as having relatively low heat tolerance (RLHT). Colonies that were not classified either as RHHT or RLHT were considered as unclassified. For brevity and ease of comprehension, we refer to RHHT colonies as ‘highs' and RLHT ones as ‘lows' in the main text but one should be clear that the terms are purely relative and pertain to the particular stress test conducted [22]. Binomial theory was used to estimate the likelihood of all fragments from an individual colony either surviving or dying by chance based on the overall mortality rate at the endpoint of the experiment (i.e. 50%; electronic supplementary material, S9).

#### (ii) Mean bleaching and mortality index

At the end of the experiment a post hoc BMI (*sensu* [24]) was calculated for each colony per sampling day as follows:

$$\mathrm{BMI}\;=\;\frac{0c_{1}\;+\;1c_{2}\;+\;2c_{3}\;+\;3c_{4}\;+\;4c_{5}}{4},$$

where *c*_1_ to *c*_5_ are the proportions of coral fragments that are in categories: apparently normal (*c*_1_), partially bleached (*c*_2_), totally bleached (*c*_3_), partially dead (*c*_4_) or fully dead (*c*_5_). A mean BMI value per colony was estimated by averaging daily BMI values. Lower BMI values indicate better health status, whereas higher BMI values indicate higher percentage of bleaching and mortality.

#### (iii) BMI profile

The aligned temperature profiles of each tank (electronic supplementary material, S10) were used to calculate a mean BMI (as described above) for the interpolated DHW data points. The resulting matrix of BMI was subjected to a *z*-score transformation (individually for each DHW data point) and visualized in a form of heatmap using heatmap.2 R package. Hierarchical clustering was used to arrange and group colonies according to their stress response profile (distance measure - Euclidean, agglomeration method-ward.D).

#### (iv) Colour and mortality index profile

The analysis described above is based on a subjective, visual assessment of fragment status. While it is relatively easy to distinguish fragment mortality, it is much harder to consistently distinguish bleaching severity using a visual assessment [70]. For this reason, we incorporated colour intensity in the analysis (figure 1*a*) using the images taken during the status monitoring. The colour intensity was calculated as a Euclidean distance from 0 in a three-dimensional space using the recorded red, green and blue (RGB) intensity values at each time point for each individual coral fragment. This metric was then used to recalculate the status assessed by the visual approach. Colour intensity was expressed as a fraction of maximum intensity (divided by the highest value measured in the experiment and therefore, contained in a range from 0 to 1) that replaced the status scores of healthy, partially bleached, and bleached (status scores 0, 1 and 2, respectively). For partial mortality, the calculation was colour intensity + 1 and dead fragments were given the maximum score of 2. These recalculated values were then used to estimate CMI or median status. The relationship between RGB values and Symbiodiniaceae density or pigment concentration (total chlorophyll) from 294 fragments was estimated to quantify the relationship between colour intensity and zooxanthellae pigment concentrations resulting in highly correlated relationships (electronic supplementary material, S8).

### (c) DNA isolation, Symbiodiniaceae ITS2 marker gene sequencing, SymPortal analysis

From each colony, a 2 cm fragment was taken from the prior to the heat stress reef and preserved in molecular grade ethanol. DNA extracts (*n* = 96 colonies) were prepared using the Qiagen DNeasy blood and tissue kit following the manufacturers protocol, including overnight proteinase K digestion. ITS2 (Symbiodiniaceae) amplicon-based sequencing was performed on the Illumina MiSeq following [71]. Briefly, the ITS2 primers SYM_VAR_5.8S2 (5′-TCGTCGGCAGCGTCAGATGTGTATAAGAGACAGGAATTGCAGAACTCCGTGAACC-3′) and SYM_VAR_REV (5′-GTCTCGTGGGCTCGGAGATGTGTATAAGAGACAGCGGGTTCWCTTGTYTGACTTCATGC-3′) (Illumina adaptors underlined) were used in a first-round PCR to amplify a approximately 450 bp fragment of the ribosomal ITS-2 region. Samples were then indexed using Illumina Nextera DNA unique dual indexes and underwent quality control using the Nanondrop One C assay and size assessment using the Tapestation 4200 D1000. The pooled equilibrated samples were run on as paired end reads on an Illumina MiSeq v3 with addition of 20% PhiX control' Cambridge, UK. ITS2 sequence data were submitted to Symportal [72] and the raw sequence reads are publicly available. We then calculated the relative abundance of different ITS2 profiles within each colony. The association of symbiont community type on RHT were tested using principal component analysis (electronic supplementary material, S4).

### (d) Quantifying variation in heat tolerance

Variation in coral heat tolerance was calculated as the difference in DHW between the more-tolerant and less-tolerant portions of the coral population (ΔDHW) at the highest level of BMI for which 95% confidence intervals were available for both portions. A conservative estimate of ΔDHW (ΔDHW_c_) was computed by comparing all coral colonies classified as either RHHT (*n* = 30 colonies) or RLHT (*n* = 31 colonies). First, the phenotypic bleaching and mortality response (BMI) was modelled as a function of DHW (continuous fixed effect), RHT category (two-level fixed effect) and their interaction using a binomial generalized linear mixed model (GLMM) [73]. Repeated measures were accounted for by allowing intercepts to vary randomly per colony. Then, ΔDHW_c_ was calculated as the difference between DHW values (mean and 95% confidence intervals) associated with a fixed BMI response level between the RHHT and RLHT groups. The BMI level used for comparison was set as close to 0.5 as possible, given the low BMI values for the modelled uncertainty of the RHHT group (many of which were still healthy at the end of the experiment).

Since ΔDHW_c_ probably underestimates the realized population-level *Δ*DHW (*Δ*DHW_p_), a further stepwise reduction in the number of colonies within each RHT group was conducted. This method involved repeating the analysis for progressively smaller sample sizes, where at each iteration, the colony closest to the population average heat tolerance (in terms of mean BMI) was removed from each RHT group ($N_{t}=N_{t-1}-1$), a new GLMM model was run, and new ΔDHW estimated. The stepwise reduction method was repeated until the upper and lower deciles of the population were reached (*n* = 10 in each RHT group), beyond which sample sizes were too low to draw conclusions.

### (e) Timing of annual bleaching–mortality conditions

The timing of onset of ABM conditions for the natal reef of the experimental colonies was estimated based on ΔDHW_p_. Future projections of DHW on coral reefs for the 0.25° grid cell surrounding Mascherchur reef were compiled from an ensemble of GCMs from the CMIP6 for two Shared Socioeconomic Scenarios (SSPs) [18]: SSP2-4.5 (24 models, meeting 150% of Paris Agreement pledges) and SSP5-8.5 (28 models, worst-case scenario, growing world economy heavily dependent on fossil fuels [27]). In these projections, future DHW was calculated as the sum of positive anomalies above the MMM for each three months in the 2015–2100 period [18]. Using these projections, we estimated the additional time that higher levels of coral heat tolerance is likely to achieve under climate change in comparison to lower levels of coral heat tolerance. The year of onset of ABM was calculated as the first year in which the entire coming decade exceeds the bleaching-mortality DHW threshold annually. To simulate differences between high and low levels of coral heat tolerance, three separate bleaching-mortality thresholds were imposed (4°C, 8°C and 12°C-weeks). These thresholds also reflect the bleaching and mortality risk levels of the NOAA Coral Reef Watch Bleaching Alert System. Differences in the timing of ABM onset among bleaching thresholds (three-level fixed effect), emissions scenarios (two-level fixed effect) and their interaction were tested using a linear mixed model (LMM) [74]. Repeated measures within each GCM were accounted for by allowing a random intercept per GCM.

## Data Availability

All R and Python code can be found at https://doi.org/10.5281/zenodo.6256164 [75], and all images for machine learning colour analysis can be found at https://doi.org/10.5281/zenodo.6256190 [76]. The data are provided in electronic supplementary material [77].

## References

[1] Poloczanska ES et al. 2016 Responses of marine organisms to climate change across oceans. Front. Mar. Sci. **3**, 62. (10.3389/fmars.2016.00062)

[2] Reed TE, Schindler DE, Waples RS. 2011 Interacting effects of phenotypic plasticity and evolution on population persistence in a changing climate. Conserv. Biol. **25**, 56-63. (10.1111/j.1523-1739.2010.01552.x) 20646016 PMC3084585

[3] Hughes AR, Inouye BD, Johnson MT, Underwood N, Vellend M. 2008 Ecological consequences of genetic diversity. Ecol. Lett. **11**, 609-623. (10.1111/j.1461-0248.2008.01179.x) 18400018

[4] Hu J, Barrett R. 2017 Epigenetics in natural animal populations. J. Evol. Biol. **30**, 1612-1632. (10.1111/jeb.13130) 28597938

[5] Nussey D, Wilson A, Brommer J. 2007 The evolutionary ecology of individual phenotypic plasticity in wild populations. J. Evol. Biol. **20**, 831-844. (10.1111/j.1420-9101.2007.01300.x) 17465894

[6] Glynn PW, D'Croz L. 1990 Experimental evidence for high temperature stress as the cause of El Niño-coincident coral mortality. Coral Reefs **8**, 181. (10.1007/BF00265009)

[7] Skirving W, Marsh B, De La Cour J, Liu G, Harris A, Maturi E, Geiger E, Eakin CM. 2020 CoralTemp and the coral reef watch coral bleaching heat stress product suite version 3.1. Remote Sensing **12**, 3856. (10.3390/rs12233856)

[8] Marshall PA, Baird AH. 2000 Bleaching of corals on the Great Barrier Reef: differential susceptibilities among taxa. Coral Reefs **19**, 155-163. (10.1007/s003380000086)

[9] Fuller ZL et al. 2020 Population genetics of the coral *Acropora millepora*: toward genomic prediction of bleaching. Science **369**, eaba4674. (10.1126/science.aba4674) 32675347

[10] Manzello DP, Matz MV, Enochs IC, Valentino L, Carlton RD, Kolodziej G, Serrano X, Towle EK, Jankulak M. 2019 Role of host genetics and heat-tolerant algal symbionts in sustaining populations of the endangered coral *Orbicella faveolata* in the Florida Keys with ocean warming. Glob. Change Biol. **25**, 1016-1031. (10.1111/gcb.14545) 30552831

[11] Ziegler M, Seneca FO, Yum LK, Palumbi SR, Voolstra CR. 2017 Bacterial community dynamics are linked to patterns of coral heat tolerance. Nat. Commun. **8**, 14213. (10.1038/ncomms14213) 28186132 PMC5309854

[12] Oliver TA, Palumbi SR. 2009 Distributions of stress-resistant coral symbionts match environmental patterns at local but not regional scales. Mar. Ecol. Progress Ser. **378**, 93-103. (10.3354/meps07871)

[13] Guest JR, Baird AH, Maynard JA, Muttaqin E, Edwards AJ, Campbell SJ, Yewdall K, Affendi YA, Chou LM. 2012 Contrasting patterns of coral bleaching susceptibility in 2010 suggest an adaptive response to thermal stress. PLoS ONE **7**, e33353. (10.1371/journal.pone.0033353) 22428027 PMC3302856

[14] Anthony K et al. 2017 New interventions are needed to save coral reefs. Nat. Ecol. Evol. **1**, 1420-1422. (10.1038/s41559-017-0313-5) 29185526

[15] van Oppen MJ et al. 2017 Shifting paradigms in restoration of the world's coral reefs. Glob. Chang. Biol. **23**, 3437-3448. (10.1111/gcb.13647) 28247459

[16] Aitken SN, Whitlock MC. 2013 Assisted gene flow to facilitate local adaptation to climate change. Ann. Rev. Ecol. Evol. Syst. **44**, 367-388. (10.1146/annurev-ecolsys-110512-135747)

[17] van Oppen MJ, Oliver JK, Putnam HM, Gates RD. 2015 Building coral reef resilience through assisted evolution. Proc. Natl Acad. Sci. USA **112**, 2307-2313. (10.1073/pnas.1422301112). 25646461 PMC4345611

[18] van Hooidonk R et al. 2020 Projections of future coral bleaching conditions using IPCC CMIP6 models: climate policy implications, management applications, and regional seas summaries. See https://www.researchgate.net/publication/346311563_Projections_of_future_coral_bleaching_conditions_using_IPCC_CMIP6_Models.

[19] Coles SL, Riegl BM. 2013 Thermal tolerances of reef corals in the Gulf: a review of the potential for increasing coral survival and adaptation to climate change through assisted translocation. Mar. Pollut. Bull. **72**, 323-332. (10.1016/j.marpolbul.2012.09.006) 23058810

[20] Quigley KM, Bay LK, van Oppen MJH. 2019 The active spread of adaptive variation for reef resilience. Ecol. Evol. **9**, 11 122-11 135. (10.1002/ece3.5616) PMC6802068 31641460

[21] Quigley KM, Bay LK, van Oppen MJH. 2020 Genome-wide SNP analysis reveals an increase in adaptive genetic variation through selective breeding of coral. Mol. Ecol. **29**, 2176-2188. (10.1111/mec.15482) 32453867

[22] Humanes A et al. 2021 An experimental framework for selectively breeding corals for assisted evolution. Front. Mar. Sci. **8**, 669995. (10.3389/fmars.2021.669995).

[23] Wall CB, Ricci CA, Wen AD, Ledbetter BE, Klinger DE, Mydlarz LD, Gates RD, Putnam HM. 2021 Shifting baselines: physiological legacies contribute to the response of reef corals to frequent heatwaves. Funct. Ecol. **35**, 1366-1378. (10.1111/1365-2435.13795)

[24] McClanahan TR. 2004 The relationship between bleaching and mortality of common corals. Mar. Biol. **144**, 1239-1245. (10.1007/s00227-003-1271-9).

[25] Lachs L, Bythell JC, East HK, Edwards AJ, Mumby PJ, Skirving WJ, Spady BL, Guest JR. 2021 Fine-tuning heat stress algorithms to optimise global predictions of mass coral bleaching. Remote Sensing **13**, 2677. (10.3390/rs13142677)

[26] McLachlan RH, Price JT, Solomon SL, Grottoli AG. 2020 Thirty years of coral heat-stress experiments: a review of methods. Coral Reefs. **39**, 885-902. (10.1007/s00338-020-01931-9)

[27] Riahi K et al. 2017 The Shared Socioeconomic Pathways and their energy, land use, and greenhouse gas emissions implications: an overview. Glob. Environ. Change **42**, 153-168. (10.1016/j.gloenvcha.2016.05.009)

[28] Brown BE, Dunne RP, Edwards AJ, Sweet MJ, Phongsuwan N. 2014 Decadal environmental ‘memory’ in a reef coral? Mar. Biol. **162**, 479-483. (10.1007/s00227-014-2596-2)

[29] Berkelmans R, van Oppen MJ.H. 2006 The role of zooxanthellae in the thermal tolerance of corals: a ‘nugget of hope’ for coral reefs in an era of climate change. Proc. R. Soc. B **273**, 2305-2312. (10.1098/rspb.2006.3567) PMC1636081 16928632

[30] Ritson-Williams R, Gates RD. 2020 Coral community resilience to successive years of bleaching in Kāne‘ohe Bay, Hawai‘i. Coral Reefs **39**, 757-769. (10.1007/s00338-020-01944-4)

[31] Cunning R, Ritson-Williams R, Gates RD. 2016 Patterns of bleaching and recovery of *Montipora capitata* in Kāne‘ohe Bay, Hawai‘i, USA. Mar. Ecol. Progress Ser. **551**, 131-139. (10.3354/meps11733).

[32] Howells EJ, Abrego D, Liew YJ, Burt JA, Meyer E, Aranda M. 2021 Enhancing the heat tolerance of reef-building corals to future warming. Sci. Adv. **7**, eabg6070. (10.1126/sciadv.abg6070) 34417178 PMC8378819

[33] Thomas L, Rose NH, Bay RA, Lopez EH, Morikawa MK, Ruiz-Jones L, Palumbi SR. 2018 Mechanisms of thermal tolerance in reef-building corals across a fine-grained environmental mosaic: lessons from Ofu, American Samoa. Front. Mar. Sci. **4**, 434. (10.3389/fmars.2017.00434).

[34] Howells EJ, Abrego D, Meyer E, Kirk NL, Burt JA. 2016 Host adaptation and unexpected symbiont partners enable reef-building corals to tolerate extreme temperatures. Glob. Chang. Biol. **22**, 2702-2714. (10.1111/gcb.13250) 26864257

[35] Thomas L, Lopez EH, Morikawa MK, Palumbi SR. 2019 Transcriptomic resilience, symbiont shuffling, and vulnerability to recurrent bleaching in reef-building corals. Mol. Ecol. **28**, 3371-3382. (10.1111/mec.15143) 31177587

[36] Barshis DJ, Ladner JT, Oliver TA, Seneca FO, Traylor-Knowles N, Palumbi SR. 2013 Genomic basis for coral resilience to climate change. Proc. Natl Acad. Sci. USA **110**, 1387-1392. (10.1073/pnas.1210224110) 23297204 PMC3557039

[37] Barott KL et al. 2021 Coral bleaching response is unaltered following acclimatization to reefs with distinct environmental conditions. Proc. Natl Acad. Sci. USA 118, e2025435118. (10.1073/pnas.2025435118) PMC8179235 34050025

[38] Morikawa MK, Palumbi SR. 2019 Using naturally occurring climate resilient corals to construct bleaching-resistant nurseries. Proc. Natl Acad. Sci. USA **116**, 10 586-10 591. (10.1073/pnas.1721415116) PMC6535031 31061118

[39] Wright RM, Mera H, Kenkel CD, Nayfa M, Bay LK, Matz MV. 2019 Positive genetic associations among fitness traits support evolvability of a reef-building coral under multiple stressors. Glob. Chang. Biol **25**, 3294-3304. (10.1111/gcb.14764) 31301206

[40] Cunning R et al. 2021 Census of heat tolerance among Florida's threatened staghorn corals finds resilient individuals throughout existing nursery populations. Proc. R. Soc. B **288**, 20211613. (10.1098/rspb.2021.1613) PMC8527199 34666521

[41] Cornwell B, Hounchell K, Walker N, Golbuu Y, Nestor V, Palumbi SR. 2020 Widespread variation in heat tolerance and symbiont load are associated with growth tradeoffs in the coral Acropora hyacinthus in Palau. eLife **10**, e64790. (10.7554/eLife.64790) PMC8457836 34387190

[42] Leggat W, Heron SF, Fordyce A, Suggett DJ, Ainsworth TD. 2022 Experiment Degree Heating Week (eDHW) as a novel metric to reconcile and validate past and future global coral bleaching studies. J. Environ. Manage. **301**, 113919. (10.1016/j.jenvman.2021.113919) 34731944

[43] Anthony KR.N., Hoogenboom MO, Maynard JA, Grottoli AG, Middlebrook R. 2009 Energetics approach to predicting mortality risk from environmental stress: a case study of coral bleaching. Functional Ecology **23**, 539-550. (10.1111/j.1365-2435.2008.01531.x)

[44] Kirk NL, Howells EJ, Abrego D, Burt JA, Meyer E. 2018 Genomic and transcriptomic signals of thermal tolerance in heat-tolerant corals (*Platygyra daedalea*) of the Arabian/Persian Gulf. Mol. Ecol. **27**, 5180-5194. (10.1111/mec.14934) 30411823

[45] Camp EF, Schoepf V, Suggett DJ. 2018 How can ‘Super Corals’ facilitate global coral reef survival under rapid environmental and climatic change? Glob Chang Biol **24**, 2755-2757. (10.1111/gcb.14153) 29582529

[46] Palmer CV. 2018 Warmer Water Affects Immunity of a Tolerant Reef Coral. Frontiers in Marine Science **5**, 253. (10.3389/fmars.2018.00253)

[47] Roach TNF, Dilworth J, Jones AD, Quinn RA, Drury C. 2021 Metabolomic signatures of coral bleaching history. Nature Ecology & Evolution. **5**, 495-503. (10.1038/s41559-020-01388-7) 33558733

[48] Howells EJ, Beltran VH, Larsen NW, Bay LK, Willis BL, van Oppen MJ.H. 2012 Coral thermal tolerance shaped by local adaptation of photosymbionts. Nat. Clim. Change **2**, 116-120. (10.1038/nclimate1330)

[49] Silverstein RN, Cunning R, Baker AC. 2015 Change in algal symbiont communities after bleaching, not prior heat exposure, increases heat tolerance of reef corals. Glob. Change Biol. **21**, 236-249. (10.1111/gcb.12706) 25099991

[50] Baker AC. 2001 Reef corals bleach to survive change. Nature **411**, 765-766. (10.1038/35081151) 11459046

[51] LaJeunesse TC, Smith RT, Finney J, Oxenford H. 2009 Outbreak and persistence of opportunistic symbiotic dinoflagellates during the 2005 Caribbean mass coral ‘bleaching'event. Proc. R. Soc. B **276**, 4139-4148. (10.1098/rspb.2009.1405) PMC2821356 19740874

[52] Hoadley KD, Lewis AM, Wham DC, Pettay DT, Grasso C, Smith R, Kemp DW, LaJeunesse TC, Warner ME. 2019 Host-symbiont combinations dictate the photo-physiological response of reef-building corals to thermal stress. Sci. Rep. **9**, 9985. (10.1038/s41598-019-46412-4) 31292499 PMC6620294

[53] Matz MV, Treml EA, Aglyamova GV, Bay LK. 2018 Potential and limits for rapid genetic adaptation to warming in a Great Barrier Reef coral. PLoS Genet. **14**, e1007220. (10.1371/journal.pgen.1007220) 29672529 PMC5908067

[54] Sully S, Burkepile DE, Donovan MK, Hodgson G, van Woesik R. 2019 A global analysis of coral bleaching over the past two decades. Nat. Commun. **10**, 1264. (10.1038/s41467-019-09238-2) 30894534 PMC6427037

[55] Cotto O, Sandell L, Chevin LM, Ronce O. 2019 Maladaptive shifts in life history in a changing environment. Am. Nat. **194**, 558-573. (10.1086/702716) 31490719

[56] Gomez-Corrales M, Prada C. 2020 Cryptic lineages respond differently to coral bleaching. Mol. Ecol. **29**, 4265-4273. (10.1111/mec.15631) 33001521

[57] Jones TA. 2013 When local isn't best. Evolutionary applications **6**, 1109-1118. (10.1111/eva.12090) 24187591 PMC3804242

[58] Jones T. 2017 Ecosystem restoration: recent advances in theory and practice. The Rangeland Journal **39**, 417-430. (10.1071/RJ17024)

[59] Moriarty T, Leggat W, Huggett MJ, Ainsworth TD. 2020 Coral Disease Causes, Consequences, and Risk within Coral Restoration. Trends Microbiol. **28**, 793-807. (10.1016/j.tim.2020.06.002) 32739101

[60] Fidelman P, McGrath C, Newlands M, Dobbs K, Jago B, Hussey K. 2019 Regulatory implications of coral reef restoration and adaptation under a changing climate. Environ. Sci. Policy **100**, 221-229. (10.1016/j.envsci.2019.04.016)

[61] Bayraktarov E, Saunders MI, Abdullah S, Mills M, Beher J, Possingham HP, Mumby PJ, Lovelock CE. 2016 The cost and feasibility of marine coastal restoration. Ecol. Appl. **26**, 1055-1074. (10.1890/15-1077) 27509748

[62] Logan CA, Dunne JP, Ryan JS, Baskett ML, Donner SD. 2021 Quantifying global potential for coral evolutionary response to climate change. Nat. Clim. Change **11**, 537-542. (10.1038/s41558-021-01037-2)

[63] van Hooidonk R, Maynard J, Tamelander J, Gove J, Ahmadia G, Raymundo L, Williams G, Heron SF, Planes S. 2016 Local-scale projections of coral reef futures and implications of the Paris Agreement. Sci. Rep. **6**, 39666. (10.1038/srep39666) 28000782 PMC5175274

[64] Bay RA, Rose NH, Logan CA, Palumbi SR. 2017 Genomic models predict successful coral adaptation if future ocean warming rates are reduced. Science Advances **3**, 1701413. (10.1126/sciadv.1701413) PMC5665595 29109975

[65] Ortiz JC, Pears RJ, Beeden R, Dryden J, Wolff NH, Gomez Cabrera MDC, Mumby PJ. 2021 Important ecosystem function, low redundancy and high vulnerability: The trifecta argument for protecting the Great Barrier Reef's tabular Acropora. Conservation Letters. **14**, e12817. (10.1111/conl.12817)

[66] Cheung MWM, Hock K, Skirving W, Mumby PJ. 2021 Cumulative bleaching undermines systemic resilience of the Great Barrier Reef. Current biology. **31**, 5385-5392. (10.1016/j.cub.2021.09.078) 34739820

[67] Bruno J, Siddon C, Witman J, Colin P, Toscano M. 2001 El Niño related coral bleaching in Palau, Western Caroline Islands. Coral Reefs **20**, 127-136. (10.1007/s003380100151)

[68] Woesik R, Houk P, Isechal AL, Idechong JW, Victor S, Golbuu Y. 2012 Climate-change refugia in the sheltered bays of Palau: analogs of future reefs. Ecol Evol **2**, 2474-2484. (10.1002/ece3.363) 23145333 PMC3492774

[69] Colin P. 2018 Ocean warming and the reefs of Palau. Oceanography **31**, 126–135. (10.5670/oceanog.2018.214)

[70] Fitt WK, McFarland FK, Warner ME, Chilcoat GC. 2000 Seasonal patterns of tissue biomass and densities of symbiotic dinoflagellates in reef corals and relation to coral bleaching. Limnology and Oceanography **45**, 677-668. (10.4319/lo.2000.45.3.0677)

[71] Voolstra CR, Buitrago-López C, Perna G, Cárdenas A, Hume BCC, Rädecker N, Barshis DJ. 2020 Standardized short-term acute heat stress assays resolve historical differences in coral thermotolerance across microhabitat reef sites. Glob. Change Biol. **26**, 4328-4343. (10.1111/gcb.15148) 32567206

[72] Hume BCC, Smith EG, Ziegler M, Warrington HJM, Burt JA, LaJeunesse TC, Wiedenmann J, Voolstra CR. 2019 SymPortal: a novel analytical framework and platform for coral algal symbiont next-generation sequencing ITS2 profiling. Mol. Ecol. Resour. **19**, 1063-1080. (10.1111/1755-0998.13004) 30740899 PMC6618109

[73] Brooks ME et al. 2017 GlmmTMB balances speed and flexibility among packages for zero-inflated generalized linear mixed modeling. R J. **9**, 378-400. (10.32614/rj-2017-066)

[74] Bates D, Mächler M, Bolker B, Walker S. 2015 fitting linear mixed-effects models using lme4. J. Stat. Softw. **67**, 1-48. (10.18637/jss.v067.i01)

[75] Lachs L, Treumann A, Palmowski P. 2022 Within population variability of coral heat tolerance – code, 1st edn. Zenodo. (10.5281/zenodo.6256164) PMC9428547 36043280

[76] Lachs L, Humanes A, Martinez HM. 2022 Within population variability of coral heat tolerance – images, 1st edn. Zenodo. (10.5281/zenodo.6256190) PMC9428547 36043280

[77] Humanes A et al. 2022 Within-population variability in coral heat tolerance indicates climate adaptation potential. *Figshare*. (10.6084/m9.figshare.c.6158545) PMC9428547 36043280

